# Enhanced Lateral Resolution Multiplane Imaging via Dynamic SLM and Microsphere Lens Control

**DOI:** 10.3390/s26144370

**Published:** 2026-07-09

**Authors:** Zhongsheng Zhai, Mingmin Liu, Zili Lei, Zhi Xiong, Da Liu, Wei Feng, Qinghua Lv, Chenwei Zhu

**Affiliations:** 1 Hubei Key Laboratory of Modern Manufacturing Quantity Engineering, School of Mechanical Engineering, Hubei University of Technology, Wuhan 430068, China; zs.zhai@hbut.edu.cn (Z.Z.); 102410068@hbut.edu.cn (M.L.); 20231136@hbut.edu.cn (Z.L.); ragbearxz@hbut.edu.cn (Z.X.); 20221030@hbut.edu.cn (D.L.); fengwei@hbut.edu.cn (W.F.); 2 School of Science, Hubei University of Technology, Wuhan 430068, China

**Keywords:** microsphere lens, phase encoding, mechanical-scan-free multiplane imaging, enhanced lateral resolution imaging

## Abstract

**Highlights:**

**What are the main findings?**

- The integration of a microsphere lens improves lateral resolution from 57.0 lp/mm to 101.6 lp/mm (78.25% increase) primarily through photonic nanojet-assisted near-field coupling and effective numerical aperture enhancement.
- Dynamic phase encoding on a spatial light modulator enables mechanical-scan-free multiplane imaging, maintaining a fixed image plane and constant magnification across axial sections.

**What are the implications of the main findings?**

- This technique provides a flexible, non-mechanical approach for enhanced-resolution 3D imaging, suitable for real-time biomedical microscopy and multilayer micro/nanofabrication.
- It overcomes conventional limitations of microsphere lenses in field of view and operational flexibility, allowing depth-resolved sample detection without physical movement or repositioning.

**Abstract:**

Microsphere lenses generate photonic nanojets with subwavelength beam waists; however, conventional imaging techniques struggle to ensure consistent quality for targets at different axial planes. An optical theory is analyzed, demonstrating that incorporating a microsphere lens not only significantly enhances resolution but also maintains uniform lateral resolution across different axial sections. In this system, a spatial light modulator (SLM) is positioned at the image-side focal plane of the front group of an infinity-corrected microscope objective, where the microsphere assists in enhancing near-field coupling and increasing the effective numerical aperture to form a new combined imaging system. Within a certain imaging range, the system can clearly image any axial section or multiple target planes without compensating for the imaging aberration of those axial sections. The lateral resolving power is improved from 57.0 lp/mm to 101.6 lp/mm (an increase of 78.25%), demonstrating enhanced capability for visualizing fine micro-scale features. This technique overcomes the limitations of conventional microsphere lenses in terms of imaging field of view and operational flexibility. In research scenarios where the sample is fixed or relative motion is undesirable, it enables the detection of sample information at different depths.

## 1. Introduction

Dielectric microsphere lenses are widely studied in high-resolution laser micro/nanofabrication because they generate photonic nanojets [1,2,3,4]. These nanojets possess subwavelength beam waists, long focal depths, and high optical intensity, enabling high-resolution nanostructure fabrication beyond conventional diffraction limits [5,6]. Laser micro/nanofabrication is essential for manufacturing microelectronic and integrated photonic devices due to its non-invasive nature, high precision, and wide material compatibility. As these devices advance toward higher-density integration and 3D stacking, an urgent need arises for technologies that enable high-resolution, sequential acquisition of different axial planes.

Many existing microsphere-assisted methods involve mechanical axial scanning. Recent studies [7] indicate that photonic nanojet characteristics depend on the particle’s Mie size parameter and the tangential electric field distribution. Moreover, photonic jets can be generated by mesoscale particles of arbitrary three-dimensional shape [8]. Furthermore, phase-sensitive particle materials have been shown to enable focal-length tuning in microsphere-based systems [9]. In non-contact laser processing, for example, repeated repositioning is needed to pattern layers at different depths [10,11]. Microsphere arrays can enable parallel processing through self-assembly, but face major limitations [12]. Pulse-induced damage and debris deposition reduce uniformity and stability. Mechanical scanning is inherently serial and point-by-point, limiting processing speed and making precise interlayer alignment difficult—especially for complex 3D devices. Although microspheres generate photonic nanojets that enable high-resolution fabrication [13,14], their potential for true 3D fabrication remains constrained by mechanical movement. Such an advancement is essential for exploiting the unique optical properties of microspheres and advancing next-generation microelectronics and photonic integration.

To address these limitations, we develop a technique for mechanical-scan-free multiplane imaging and potential fabrication by synergistically integrating an SLM and a dielectric microsphere lens. This optical architecture differs from prior SLM-based multiplane systems by incorporating a microsphere in the object path to enhance lateral resolution. The SLM was positioned at the image-side focal plane of the front group of an infinity-corrected microscope objective [15,16]. Dynamic SLM-based phase encoding enabled flexible switching among multiple axial planes without physical movement, while the microsphere lens generated photonic nanojets with subwavelength beam widths and enhanced near-field intensity, effectively improving lateral resolution [14,17,18]. By combining programmable multi-plane modulation with near-field enhancement, stable high-resolution imaging and fabrication across multiple object planes were achieved.

The optical design incorporated a tunable focusing module at the image-side focal plane of the infinity-corrected objective, enabling multi-plane imaging with a stationary image plane. Full-wave simulations based on the three-dimensional finite-difference time-domain (FDTD) method were employed to analyze the photonic nanojet effect and its impact on resolution. An imaging system consisting of an objective, SLM, relay optics, and a microsphere module was constructed. Using customized phase patterns, resolution enhancement and layered multi-plane imaging were demonstrated without mechanical scanning, confirming the system’s capability for scan-free, high-resolution multi-plane imaging.

## 2. Experiment

### 2.1. Instrumental Setup

**Figure 1:**
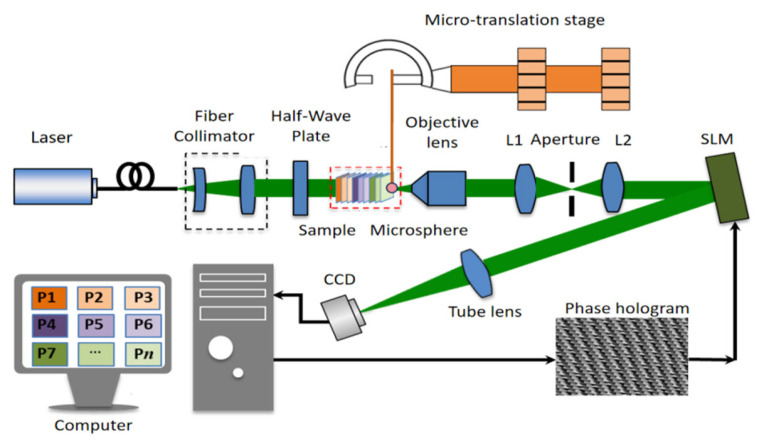
Schematic of the proposed mechanical-scan-free multiplane imaging system.

The proposed SLM projection-based high-resolution multi-plane imaging system was designed, as shown in Figure 1.

A single-mode fiber-coupled laser (AUT-FCL-655-80 T, 655 nm ± 5 nm, manufactured by Shenzhen Yimai Optoelectronics Co., Ltd. in Shenzhen, China) with a 2 mm collimated spot provided uniform illumination. The beam passed through a collimating and expanding system consisting of a 190 mm focal length lens and a pinhole filter made of a 10× infinite tube-length objective and a 25 µm pinhole to improve spot uniformity. A half-wave plate adjusted the laser polarization to match the liquid crystal alignment of the Hamamatsu X13138 reflective spatial light modulator (1280 × 1024 pixels, 12.5 µm pitch, manufactured by Hamamatsu Photonics K.K. in Hamamatsu, Japan). A PMMA microsphere lens (radius: 500 µm, n = 1.49) was placed between the objective and the resolution target. Light from the sample was collected by a 10× objective ($f_{\mathrm{obj}}$ = 20 mm), then transmitted through a 4f relay system (L1 and L2, $f_{1}$ = $f_{2}$ = 110 mm), aligning the objective’s image-side focal plane with the SLM surface. By applying digital phase masks to the SLM, a new imaging system was formed with the objective front group and an integrated microsphere lens, achieving multiplane object-image conjugation. Finally, imaging beams from different axial planes were focused via a 75 mm imaging lens onto a CCD camera (1280 × 1024 pixels, 4 µm pitch).

### 2.2. Methods

This study presented a mechanical-scan-free multi-plane imaging method using an SLM and a microsphere lens, validated through theoretical modeling, full-wave simulation, and experiments. A thick-lens model of the microsphere was used to determine its focal position, magnification, and refractive index relationship. Leveraging infinity-corrected optics, an optical system was designed with a tunable focus module placed at the objective’s image-side focal plane, enabling multi-plane imaging with a fixed image plane. Full-wave electromagnetic simulations of the microsphere’s photonic nanojet effect were performed using a 3D FDTD method, with near-to-far-field transformation extending the computational domain to the far field for quantitative resolution analysis.

In the experimental phase, an imaging system was constructed using an infinity-corrected objective, an SLM, a Fourier relay lens pair, and a microsphere module. Single-focus and multiplexed lens phase patterns were designed and loaded onto the SLM. Two key experiments were conducted: (1) a quantitative resolution test with a United States Air Force (USAF) 1951 resolution target where phase masks of different focal lengths were sequentially applied to image multiple axial sections without moving the image plane, enabling measurement of lateral resolution improvement; and (2) a multi-plane layered imaging experiment using a custom two-glass-slide sample, in which dynamic switching of SLM phase patterns allowed selective focusing and sequential imaging of different object planes. The optical configuration follows a Fresnel-zoneplate-like focusing model [19].

## 3. Theoretical Analysis

### 3.1. Imaging Model and Analysis of Microsphere Lenses

**Figure 2:**
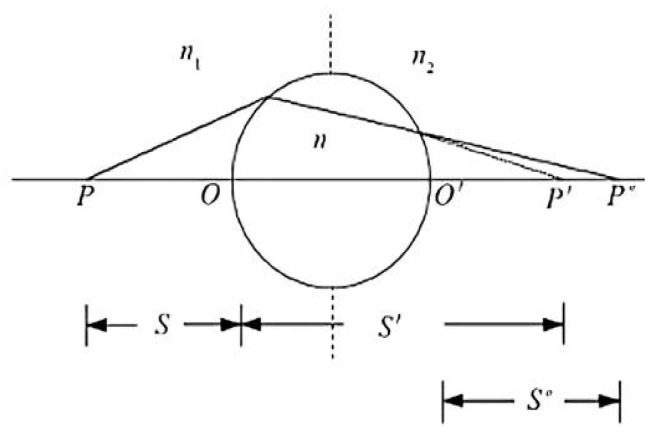
Schematic of focal position calculation for a transparent dielectric microsphere.

Transparent dielectric microspheres can be analyzed using a thick-lens model (Figure 2), which is based on sequential refraction at two spherical interfaces and allows accurate determination of the focal position. Here, n denotes the refractive index of the microsphere, while $n_{1}$ and $n_{2}$ represent the refractive indices of the surrounding media on either side. The centers of curvature are labeled O and $O^{\prime }$. A point source P produces an intermediate image $P^{\prime }$ at the first surface, which is subsequently imaged as $P^{\prime \prime }$ at the second surface. According to geometrical optics, S and $S^{\prime \prime }$ denote the object and final image distances, whereas S′ and S′-2r correspond to the image distance of the first surface and the conjugate object distance for the second surface, respectively, with r being the microsphere radius.

Based on the principles of spherical refraction, the optical propagation characteristics of a transparent microsphere can be expressed by Equations (1) and (2):

(1) $$\frac{n}{s^{\prime }}+\frac{n_{1}}{s}\;=\;\frac{n-n_{1}}{r}\;\;$$

(2) $$\frac{n_{2}}{s^{\prime \prime }}-\frac{n}{s^{\prime }-2r}=\frac{n_{2}-n}{-r}\;\;$$

For parallel incident light (S = ∞), the image distance can be determined from Equations (3)–(5):

(3) $$s^{\prime }\;=\;\frac{nr}{n-n_{1}}\;\;$$

(4) $$s^{\prime \prime }=\frac{n_{2}r(2n_{1}-n)}{nn_{1}+nn_{2}-2n_{1}n_{2}}\;\;$$

(5) $$f^{\prime }=s^{\prime \prime }=\frac{n_{2}r(2n_{1}-n)}{nn_{1}+nn_{2}-2n_{1}n_{2}}\;$$

here, $f^{\prime }$ denotes the distance from the focal point to $O^{\prime }$. When $n_{1}\;={\;n}_{2}$, $f^{\prime }$ is given by Equation (6):

(6) $$f^{\prime }\;=\;\frac{\left(2n_{1}-n\right)r}{2\left(n-n_{1}\right)}\;\;$$

when $n_{1}\;=\;n_{2}\;=\;1$, the distance from the focal point to the microsphere center is given by Equation (7):

(7) $$f\;=\;f^{\prime }+r\;=\;\frac{\left(2-n\right)r}{2\left(n-1\right)}+r\;=\;\frac{nr}{2\left(n-1\right)}$$

**Figure 3:**
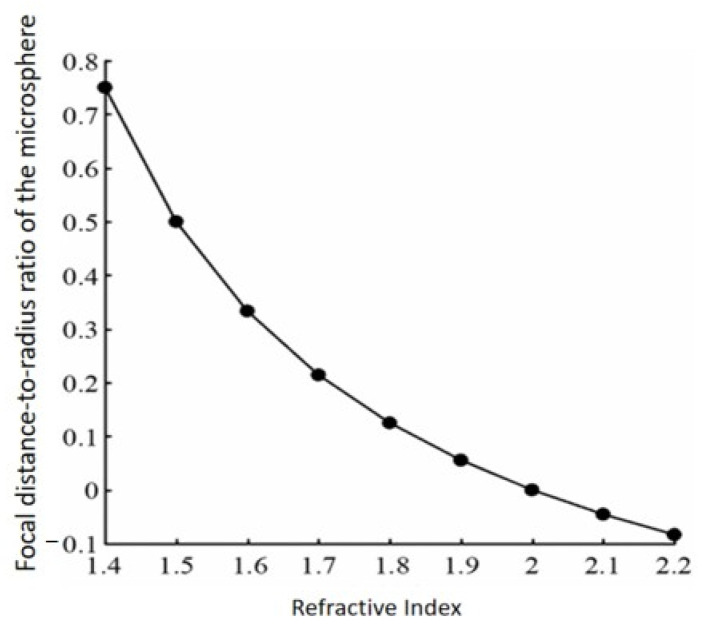
Variation in microsphere focal position with refractive index.

Figure 3 quantifies the relationship between the microsphere’s relative focal position (f/r) and its refractive index (n). As n increases, f/r decreases nonlinearly, while the focal divergence angle (θ) expands, substantially enhancing the numerical aperture (NA = n sinθ)—the fundamental mechanism enabling enhanced lateral resolution imaging. Notably, when n = 2, the focus lies exactly at the microsphere surface; for n > 2, it shifts inward. This distinctive focusing behavior enables high-index microspheres to collect and redirect high-spatial-frequency optical information, thereby enhancing imaging performance. Experimentally observed improvements are primarily due to effective numerical aperture enlargement, virtual-image magnification, and partial near-field coupling, consistent with the analysis of Darafsheh [20].

The magnification properties of transparent dielectric microspheres primarily depend on the relative positioning of the object and the focal point. When the imaging condition satisfies an object distance smaller than the focal length, the system produces a virtual image, with the corresponding magnification given by:

Here, M denotes the system magnification and u the object distance. As the refractive index n increases, the focal length f decreases, leading to a reduction in magnification m if u is held constant. Conversely, for a fixed n, increasing the object distance u results in a higher magnification.

(8) $$M=\frac{f}{f-u}\;\;$$

For a real image, the magnification is given by Equation (9):

(9) $$M\;=\;\frac{f}{u-f}\;\;$$

### 3.2. Optical Design for Scan-Free Multiplane Imaging

**Figure 4:**
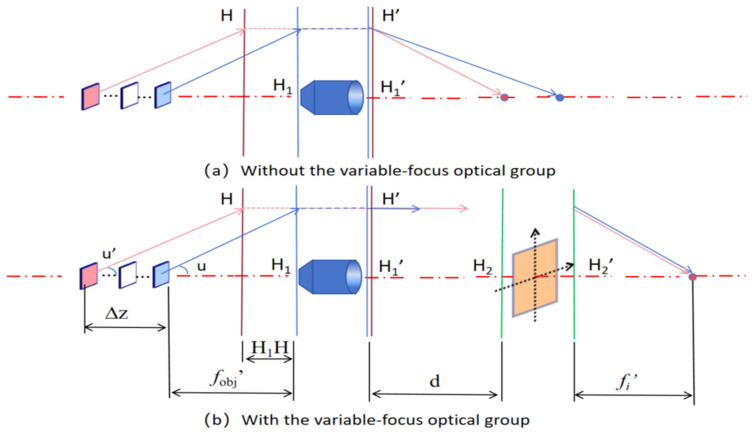
Optical layout for multi-plane imaging with preserved image quality.

In this study, an infinity-corrected microscope forms the foundational architecture, into which a tunable-focus optical module is integrated behind the objective front group to construct a compound imaging system, as illustrated in Figure 4. The design’s key parameters include the front-group focal length of the objective ${f_{\mathrm{obj}}}^{\prime }$, the focal lengths of the tunable optical groups (${f_{i}}^{\prime }(i\;=\;1,2,\cdots ,n)$), and the inter-component spacing d. The system’s principal planes are designated as $H_{1}$ and ${H_{1}}^{\prime }$ for the object- and image-side planes of the objective, and $H_{2}$ and ${H_{2}}^{\prime }$ for the object- and image-side planes of the tunable module. This modular design preserves the advantages of an infinity-corrected system while enabling flexible focal-length adjustment.

Based on geometric optics, the effective focal length of the compound system is given by:

(10) $$f^{\prime }\;=\;\frac{f_{obj}^{\prime }f_{i}^{\prime }}{f_{obj}^{\prime }+f_{i}^{\prime }-d}\;$$

We adopt the analytical expression for object-plane shifts from [21] (Equation (9)), as it accurately describes that the image plane remains fixed while the object plane shifts with changes in $f_{i}^{\prime }$, with the displacement equal to that of the system’s object-side principal plane:

(11) $$∆z\;=\;\frac{{\left(f_{obj}^{\prime }\right)}^{2}}{f_{i}^{\prime }}\;\;$$

Equation (11) is derived under the paraxial approximation and assumes that the SLM phase modulation can be represented by an equivalent thin lens. Aberrations arising from pixel discretization and phase quantization are neglected in this analytical model.

This optical system integrates an imaging group with variable focal lengths at the image-side focal plane of the objective front group, enabling sequential electronic refocusing of multiple sample planes while maintaining constant magnification and a fixed image plane. Since the aperture stop and field stop remain unchanged, the field of view is preserved across all imaging depths. By applying $u\;=\;u^{\prime }$ and the numerical aperture formula NA = nsinu, the design further guarantees consistency of numerical aperture and field of view in multi-plane imaging.

## 4. Results and Discussion

### 4.1. Simulation

**Figure 5:**
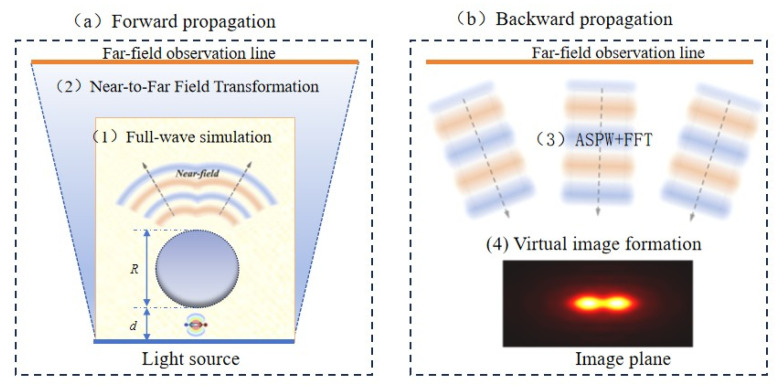
Simulation architecture. (**a**) Forward propagation. (1) full-wave simulation and (2) near-to-far-field (NTFF) transformation. (**b**) Backward propagation. (3) angular spectrum of plane waves (ASPW) via fast Fourier transform (FFT) and (4) virtual image formation.

A simulation framework based on wave optics and inspired by analogous concepts was established, as illustrated in Figure 5. The architecture consisted of two primary modules: forward propagation and backward propagation. Each module was divided into the following processes: (1) full-wave simulation, (2) near-to-far-field (NTFF) transformation for forward propagation, (3) angular spectrum of plane waves (ASPW) implemented via fast Fourier transform (FFT), and (4) virtual image formation for backward propagation. Since the main objective of the study was to evaluate the impact of microsphere integration on resolution, the simulation focused primarily on the NTFF stage.

#### 4.1.1. 3D FDTD Full-Wave Modeling

**Figure 6:**
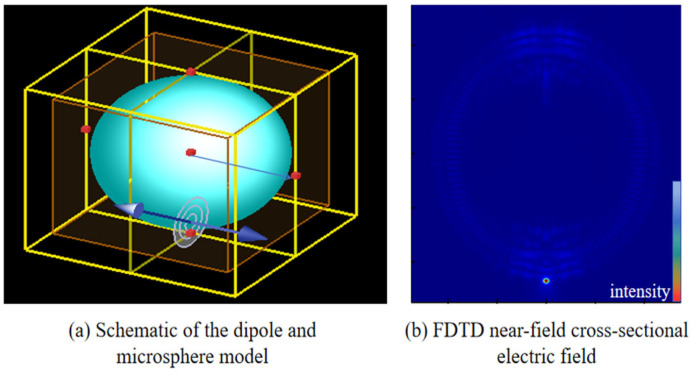
FDTD-based microsphere model and simulated near-field electric field distribution. (**a**) FDTD model of a 5 μm radius microsphere (n = 1.46) with a dipole source 200 nm below the surface. (**b**) Simulated near-field cross-sectional electric field; the color bar indicates relative intensity.

In this study, the FDTD method was selected as the simulation platform. A uniform mesh with a grid size of 20 nm (approximately λ/32 at 655 nm) was employed in the region near the microsphere and the object plane. Perfectly matched layer (PML) boundary conditions were applied with a thickness of 1 μm, and the total-field/scattered-field (TFSF) source was used to introduce a plane wave. To prevent premature absorption of evanescent waves, the PML boundaries were maintained at a distance greater than half a wavelength from the microsphere surface. Due to computational resource constraints, the 3D-FDTD simulation region was kept within 10 μm. Electric and magnetic fields were recorded on all six boundaries of the simulation region for subsequent near-to-far-field transformation. The focusing efficiency of the microsphere, defined as the ratio of optical power concentrated within the nanojet region to the total incident power, was qualitatively evaluated from the simulated field distribution. The strong field localization observed behind the microsphere indicates efficient optical concentration. Since the present work focuses primarily on imaging performance, a detailed quantitative efficiency analysis is beyond the scope of this study. Figure 6 illustrates the microsphere model and the corresponding electric field distribution obtained from the FDTD simulation.

#### 4.1.2. Resolution Evaluation via Near-to-Far-Field Transformation

**Figure 7:**
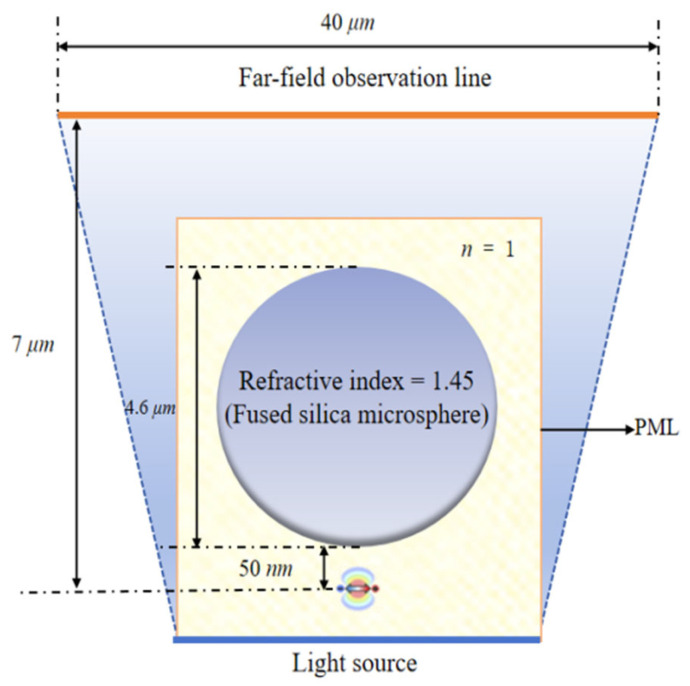
Schematic of the simulation with fused-silica microspheres.

**Figure 8:**
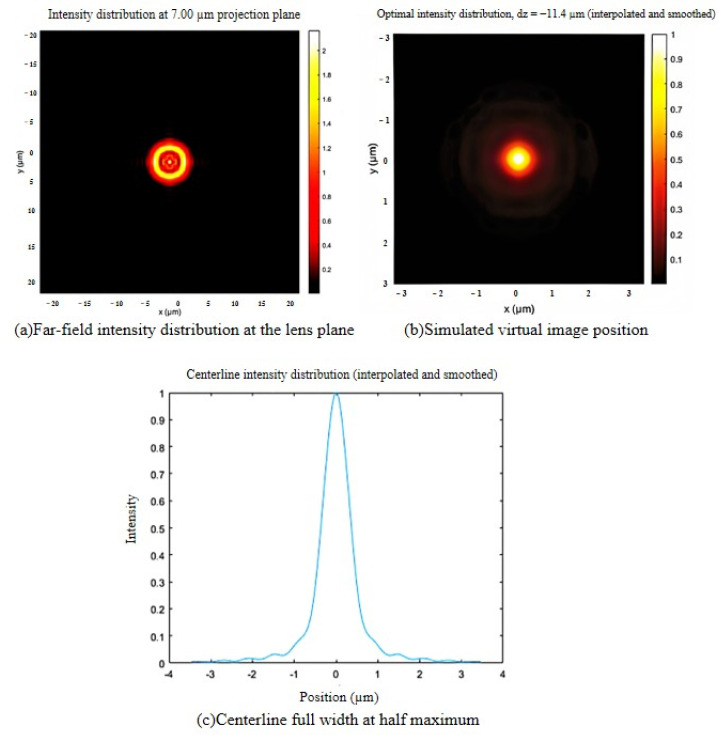
Microsphere parameters based on FDTD simulation.

The model was applied to simulate experiments with fused-silica microspheres, as shown in Figure 7 A 40 × 40 μm monitoring region was placed above the object plane, extending the computational domain laterally by nearly an order of magnitude. Key parameters matched experimental conditions: an object–projection plane distance of 7 μm (collection angle ~140°), yielding a numerical aperture of 0.94 from the relation NA = n sinθ. The image plane $Z_{f}$ was normalized to the object plane, and the wavelength was fixed at 400 nm for spectral consistency. As shown in Figure 8, this boundary treatment improves computational efficiency while ensuring comparability with experimental results.

To quantitatively evaluate the resolving capability of the microsphere-assisted system, we adopt the effective resolution criterion defined as ∆l≅d/M, where d is the full-width at half-maximum (FWHM) of the point spread function (PSF) in the virtual image plane and M is the lateral magnification. Using this criterion in our FDTD-based simulation framework, with the image plane positioned at $z_{f}=-4.45\;µm$, the system exhibits a lateral magnification of 2.9, which agrees well with the experimental value reported in [22] (their simulated value was 3.1, corresponding to a deviation of approximately 6.5%). The simulation predicts a strongly confined focal spot corresponding to an effective object-plane resolution on the order of several hundred nanometers, which is consistent with values reported in previous microsphere-assisted imaging studies. Furthermore, a parameter sensitivity analysis demonstrates that increasing the image-to-object-plane distance to 9 μm leads to a systematic increase in magnification, while the effective resolution remains stably converged at approximately 240 nm. This convergence confirms that the resolution improvement observed in experiments primarily arises from microsphere-induced numerical aperture enlargement and near-field coupling, rather than purely from magnification effects.

### 4.2. Quantitative Imaging Analysis Using a Resolution Target

**Figure 9:**
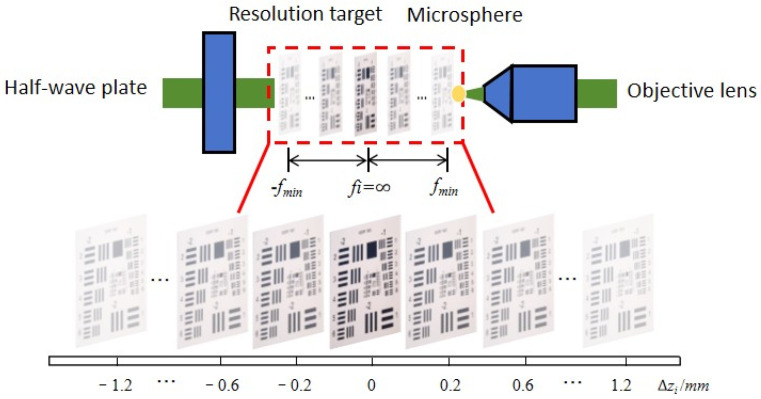
Schematic of the imaging system with an inserted resolution target.

A PMMA microsphere lens (radius: 500 µm, n = 1.49) was placed between the objective and the resolution target. As shown in Figure 9, system lateral resolution was evaluated using a USAF 1951 test chart. The axial focus was adjusted to compensate for the microsphere-induced focal shift until the sharpest image was achieved on the CCD.

**Figure 10:**
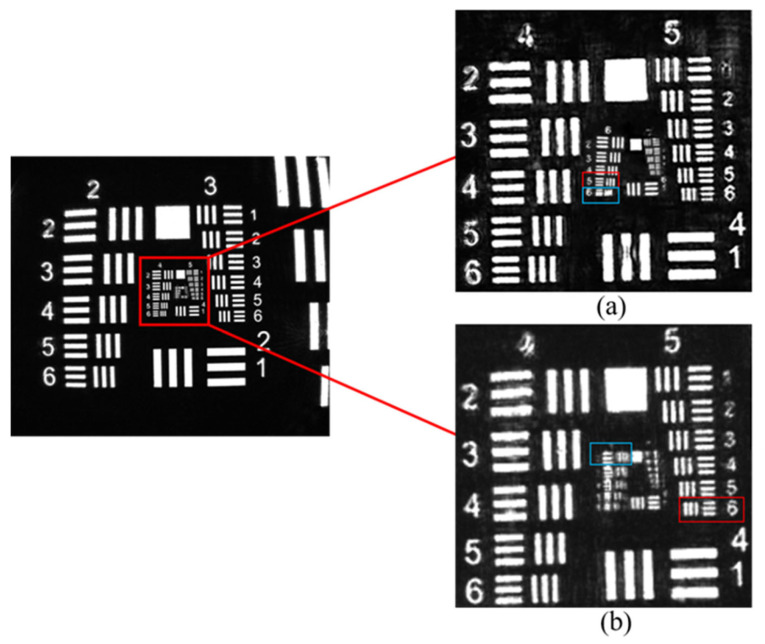
Imaging results of the original microscopic system’s inherent imaging object plane resolution. (**a**) Imaging results after adding microsphere lenses to the system. (**b**) Imaging results without adding microsphere lenses.

As shown in Figure 10, the imaging performance comparison shows: (a) the system with the microsphere lens resolves Group 6, Element 6 of the USAF target (101.6 lp/mm) in both horizontal and vertical directions; (b) the system without the microsphere lens only resolves Group 5, Element 5 (57.0 lp/mm). The improvement from 57.0 to 101.6 lp/mm represents a 78.25% increase in lateral resolving power. This enhancement is attributed to the microsphere’s contribution to effective numerical aperture, virtual-image magnification, and the redirection of high-angle scattered light into the collection path of the objective. The red line indicates the finest discernible line pair, while the blue line marks the coarsest undiscernible line pair [21].

Based on the specific parameters of the SLM, the minimum focal length achievable for the multiplexed lens is calculated to be $f_{\min}$ = 306 mm. When the focal length $f_{i}$ of the multiplexed lens is set to $\pm {\;f}_{\min}$, the corresponding imaging range of the system is $I_{\mathrm{range}}\;$= −1.31 mm~1.31 mm.

To analyze the effect of newly added optical groups on lateral resolution, multiple test sections were uniformly arranged along the optical axis, using the system’s original imaging plane as reference. The focal length fi of each section was determined using Equation (11). Table 1 illustrates the displacement relationship between the image plane and the optical groups loaded with different $f_{i}$ values.

**Figure 11:**
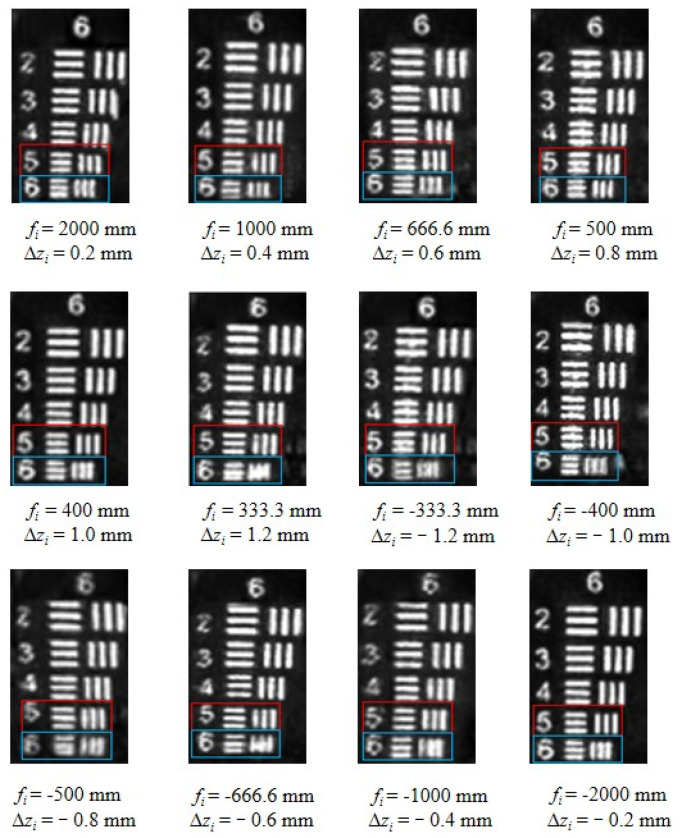
Imaging results of resolution target at different axial sections.

Single-lens phase masks with focal lengths $f_{i}$ were sequentially loaded onto the SLM. The axial knob of a precision translation stage was then adjusted, and the CCD was used to capture images at multiple axial sections. The experimental results are shown in Figure 11.

As shown in Figure 11, the red markers indicate the minimum resolvable line pairs of the system, while the blue markers correspond to line pairs beyond its resolution limit. The experimental results reveal a progressive decline in imaging quality with increasing axial displacement. In particular, when a single-lens phase mask with a focal length of $f_{i}$ = 333.3 mm was applied, the sixth group, sixth element of the resolution target exhibited pronounced horizontal blurring compared with other imaging results.

Similarly, within the imaging range of ${I_{\mathrm{range}}}^{\prime }\;=\;$−1.0 mm to 1.0 mm, the system exhibits optimal performance. Beyond ±1.0 mm, image quality gradually deteriorates due to increased wavefront aberrations and reduced alignment tolerance. Therefore, ±1.0 mm represents the practical operating range with nearly constant lateral resolving power. By adjusting the focal length parameters of the optical group, the lateral resolution across axial sections remains constant, reaching 101.6 lp/mm in both horizontal and vertical directions. Compared with the resolution of 57.0 lp/mm without the microsphere lens [21], a significant improvement is achieved.

### 4.3. Time-Sequential Multi-Plane Imaging via Electronic Refocusing

**Figure 12:**
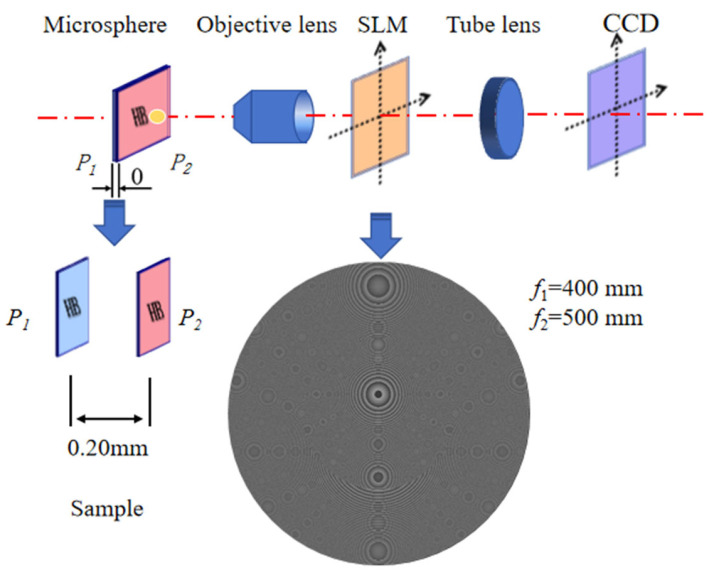
Schematic diagram of the experimental setup.

**Figure 13:**
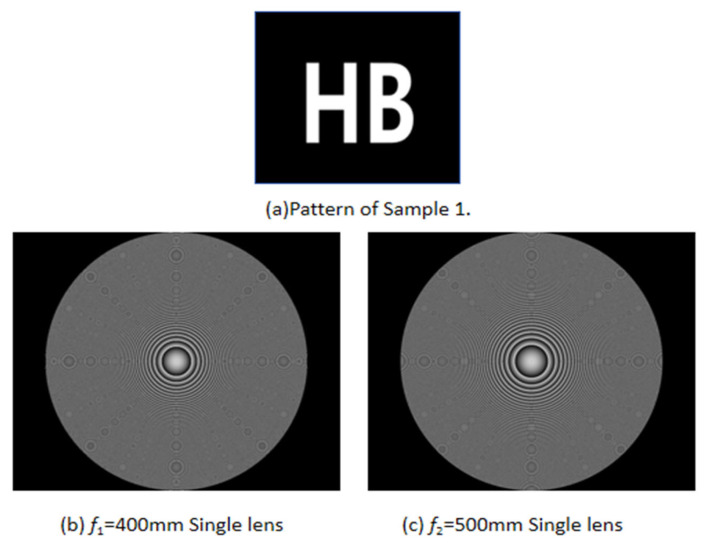
Phase masks of single-focus Fresnel lenses. (**a**) Test pattern printed on each glass slide (Sample 1). (**b**) Fresnel lens phase mask with focal length $f_{1}=400\;mm$. (**c**) Fresnel lens phase mask with focal length $f_{2}=500\;mm$.

A schematic of the experimental setup is presented in Figure 12. The sample consisted of two glass slides (each ~1 mm thick) separated by a gap of 0.20 mm. The identical test patterns shown in Figure 13a were fabricated on the surface of each slide, and the assembly was mounted on a micro-translation stage. Independent micro-translation modules equipped with PMMA microsphere lenses were deployed in parallel within the optical path. Before data acquisition, the translation stages were used solely for initial system alignment: the axial and lateral positions were adjusted once to set a constant microsphere-to-sample distance, ensure uniform illumination of the sample center, and establish coaxial alignment of the optical system. As illustrated in Figure 13b,c, Fresnel lens phase masks with focal lengths $f_{1}$ = 400 mm and $f_{2}$ = 500 mm were sequentially loaded onto the SLM, and single-focus images were captured using a CCD to evaluate the system’s optical performance for different focal lengths.

**Figure 14:**
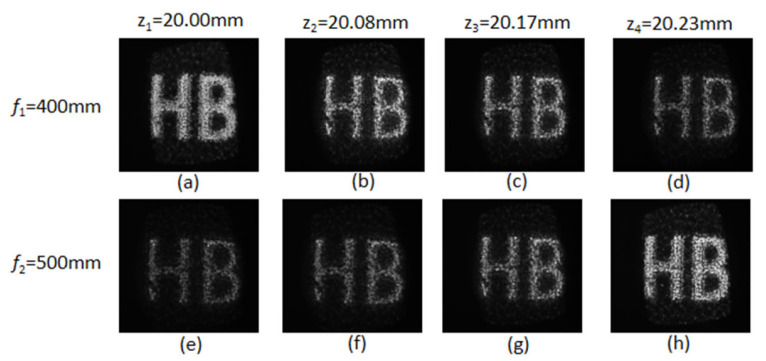
Single-plane imaging results. (**a**) Sharp image at z_1_ = 20.00 mm with f_1_ = 400 mm. (**b**) Blurred at z_2_ = 20.08 mm with f_2_ = 400 mm. (**c**) Further blurred at z_3_ = 20.17 mm with f_1_ = 400 mm. (**d**) Severely blurred at z_4_ = 20.23 mm with f_1_ = 400 mm. (**e**) Blurred at z_1_ = 20.00 mm with f_2_ = 500 mm. (**f**) Partially recovered at z_2_ = 20.08 mm with f_2_ = 500 mm. (**g**) Further recovered at z_3_ = 20.17 mm with f_2_ = 500 mm. (**h**) Sharp image at z_4_ = 20.23 mm with f_2_ = 500 mm.

When the single-focus phase mask $f_{1}=400\;mm$ was loaded, the sample and microsphere stages were synchronously adjusted to maintain a constant microsphere–sample distance until the sharpest CCD image was obtained. The object-plane position of this first conjugate relationship was $z_{1}=20.00\;mm$ (Figure 14a). Switching to $f_{2}=500\;mm$ disrupted conjugation and produced a blurred image (Figure 14e). The stage was then readjusted to recover a sharp image, yielding $z_{2}=20.23\;mm$ for the second conjugate relationship (Figure 14h). Reloading $f_{1}=400\;mm$ at $z_{4}$ again produced a blurred image (Figure 14d). These results confirm a strict one-to-one correspondence between object-plane position and focal length, demonstrating time-sequential acquisition of different axial planes via dynamic phase-mask switching. This implementation provides electronic refocusing without physical movement.

**Figure 15:**
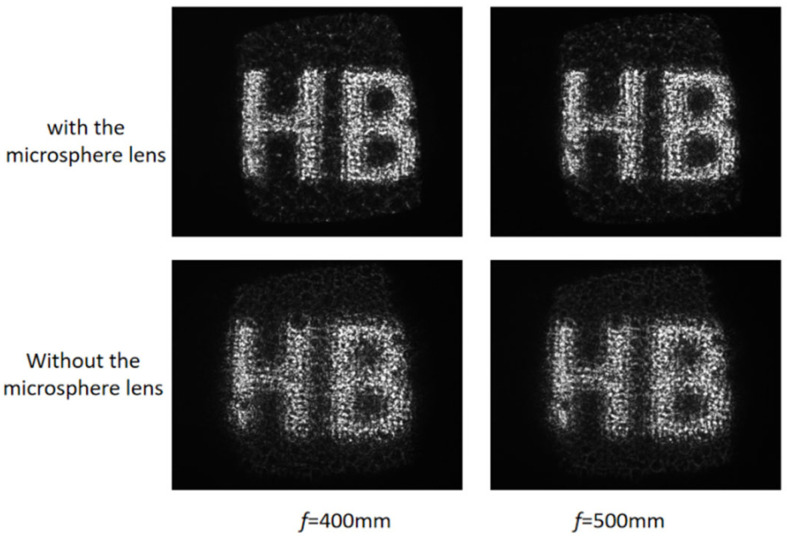
Effect of the microsphere lens on imaging.

In the experiment, the microsphere lens module was removed from the optical path, and Fresnel lens phase masks with focal lengths $f_{1}\;$= 400 mm and $f_{2}\;$= 500 mm were sequentially loaded onto the SLM. The same procedure was repeated to obtain the sharpest images within the theoretical range, which were then compared with those in Figure 14. As shown in Figure 15, under identical Fresnel lens phase mask conditions, the system equipped with the microsphere lens module exhibited markedly improved imaging performance compared to the system without the microsphere lens.

The feasibility of microsphere-lens-based multiplane imaging with enhanced resolving capability was experimentally demonstrated. With a fixed image plane and dynamic phase mask updates on a SLM, the system achieved sequential enhanced-resolution imaging across multiple axial planes.

## 5. Discussion

Compared to representative SLM-based multiplane imaging systems [21,23,24], the proposed architecture introduces a microsphere in the object path to enhance lateral resolution, a feature typically not addressed in pure SLM refocusing systems. Conversely, relative to standard microsphere-assisted imaging setups [12,15], the integration of an SLM at the Fourier plane of an infinity-corrected objective enables electronic axial scanning without mechanical sample or objective movement. This synergistic combination results in a system that offers both enhanced resolution and flexible, non-mechanical depth selection, addressing limitations of each constituent technique when used in isolation.

Compared to existing microsphere-based methods, this approach enables scan-free, sequential multi-plane imaging while maintaining consistent lateral resolution across the working depth of field. As shown in Table 2, previous techniques such as mechanical axial scanning with microsphere arrays [12] or AFM-based positioning for point-by-point writing achieve around 290 nm features but suffer from low throughput [25]. These methods rely on sequential movement, which reduces imaging speed and alignment precision and introduces instability due to mechanical vibration and contamination risks [10,11]. In contrast, the SLM-driven method allows immediate switching between axial planes without physical motion, avoiding repeated adjustments and improving both stability and speed.

By integrating a high-NA microsphere lens with programmable SLM modulation, the system achieves a 78.25% improvement in lateral resolving power (from 57.0 to 101.6 lp/mm) while maintaining a fixed image plane and nearly constant magnification across the axial scanning range of ±1 mm. Importantly, the system ensures uniform magnification and field of view across all imaging planes, a key advantage over earlier systems where variations in sphere arrangement [11] or contact conditions during scanning [12,26] lead to inconsistency.

The temporal performance of the proposed system is primarily limited by the refresh characteristics of the SLM. Since focal-plane switching is achieved through sequential loading of phase masks, the current implementation is most suitable for static or slowly varying samples. Future improvements could employ high-speed SLMs or alternative programmable optical elements to further enhance volumetric imaging speed. Further resolution enhancement may be achieved by optimizing the microsphere profile or using aspheric designs [25]. Recent work has established the functional equivalence between a microlens and a metalens for certain imaging tasks [26], suggesting potential for further system integration.

This work presents a versatile, high-throughput imaging platform that combines microsphere-assisted resolution enhancement with dynamic multi-plane imaging, suitable for real-time 3D microscopy and multi-layer micro or nano fabrication.

## 6. Conclusions

A scan-free multiplane imaging method with enhanced lateral resolution was developed by integrating an SLM with a microsphere lens. The system enables improved lateral resolving power across multiple axial planes without mechanical scanning, while maintaining a fixed image plane and nearly constant magnification. Experimental results showed that the microsphere lens improved lateral resolution from 57.0 lp/mm to 101.6 lp/mm, representing a 78.25% improvement. Multiplane imaging was achieved by applying customized phase masks on the SLM, allowing sequential focusing on different object planes without physical movement. Full-wave FDTD simulations confirmed that photonic nanojets play a key role in local field enhancement and effective numerical aperture increase, which contribute to the observed improvement in lateral resolving power. This method provides a flexible, non-mechanical approach for enhanced-resolution 3D imaging and may serve as a useful platform for future applications in biomedical imaging and multi-layer micro/nanofabrication. Future research could focus on imaging dynamic samples, improving speed through optimized SLM patterns, and incorporating adaptive optics for aberration correction in thick samples.

## Figures and Tables

**Table 1:** Relationship between the focal length of the added optical system and the axial displacement of the object plane.

$f_{i}$/mm	2000	1000	666.7	500	400	333.3
$∆z_{i}$/mm	0.2	0.4	0.6	0.8	1.0	1.2
$f_{i}$/mm	−2000	−1000	−666.7	−500	−400	−333.3
$∆z_{i}$/mm	−0.2	−0.4	−0.6	−0.8	−1.0	−1.2

**Table 2:** Comparison of the proposed method with representative SLM-based multiplane imaging and microsphere-assisted systems.

Feature	Ref. [21] (SLM Only)	Refs. [22,25] (Microsphere Only)	This Work
Lateral resolution (lp/mm)	57.0	~290 nm features	101.6
Mechanical scanning	No	Yes	No
Multiplane capability	Yes (sequential)	No (single plane)	Yes (sequential)
Fixed magnification across planes	Yes	N/A	Yes
Near-field enhancement	No	Yes	Yes

## Data Availability

Data underlying the results presented in this paper are not publicly available but may be obtained from the authors upon reasonable request.

## Abbreviations

The following abbreviations are used in this manuscript:

SLM	Spatial light modulator
FDTD	Finite-difference time-domain
CCD	Charge-coupled device
USAF	United States Air Force
NTFF	Near-to-far-field
ASPW	Angular spectrum of plane waves
FFT	Fast Fourier transform
PML	Perfectly matched layer
PMMA	Poly(methyl methacrylate)
NA	Numerical aperture
AFM	Atomic force microscope
